# A Bifunctional Anti-PD-1/TGF-β Fusion Antibody Restores Antitumour Immunity and Remodels the Tumour Microenvironment

**DOI:** 10.3390/ijms26157567

**Published:** 2025-08-05

**Authors:** Lidi Nan, Yuting Qin, Xiao Huang, Mingzhu Pan, Xiaomu Wang, Yanqing Lv, Annette Sorensen, Xiaoqiang Kang, Hong Ling, Juan Zhang

**Affiliations:** 1 Antibody Engineering Laboratory, School of Life Science and Technology, China Pharmaceutical University, Nanjing 211100, China; 2 Nanjing Leads Biolabs Co., Ltd., Nanjing 210019, China; 3 Strathclyde Institute of Pharmacy and Biomedical Sciences, University of Strathclyde, Glasgow G1 1XQ, UK

**Keywords:** bifunctional fusion antibody, PD-1, TGF-β, cancer immunotherapy

## Abstract

Although PD-1/PD-L1 inhibitors have transformed cancer immunotherapy, a substantial proportion of patients derive no clinical benefit due to resistance driven by the tumour microenvironment (TME). Transforming growth factor-β (TGF-β) is a key immunosuppressive cytokine implicated in this resistance. Several bifunctional antibodies that co-target PD-1 and TGF-β signalling have entered clinical trials and shown encouraging efficacy, but the mechanistic basis of their synergy is not fully understood. Here, we engineered 015s, a bifunctional fusion antibody that simultaneously targets murine PD-1 and TGF-β and evaluated its antitumour efficacy and mechanistic impact in pre-clinical models. Antibody 015s exhibited high affinity, dual target binding, and the effective inhibition of PD-1 and TGF-β signalling. In vivo, 015s significantly suppressed tumour growth compared with anti-mPD-1 or TGF-β receptor II (TGF-βRII) monotherapy. When combined with the CD24-targeted ADC, 015s produced even greater antitumour activity and achieved complete tumour regression. Mechanistic studies demonstrated that 015s significantly reduced tumour cell migration and invasion, reversed epithelial–mesenchymal transition (EMT), decreased microvascular density, and attenuated collagen deposition within the TME. Antibody 015s also decreased bioactive TGF-β1 and increased intratumoural IFN-γ, creating a more immunostimulatory milieu. These findings support further development of PD-1/TGF-β bifunctional antibodies for cancers with high TGF-β activity or limited response to immune checkpoint blockade.

## 1. Introduction

Immune checkpoints are pivotal modulators of antitumour immunity, and cancer cells routinely co-opt these pathways to suppress immune responses and evade host surveillance. Consequently, monoclonal antibodies that block the PD-1/PD-L1 and CTLA-4 checkpoints have revolutionised cancer therapy [1].

Engagement of programmed cell death protein-1 (PD-1) by its ligand PD-L1 transmits a co-inhibitory signal that suppresses T cell-mediated antitumour responses and enables immune escape [2,3,4]. Therapeutic antibodies that disrupt the PD-1/PD-L1 axis restore T cell activity and have shown clinical benefits in melanoma [5,6,7,8], non-small-cell lung cancer [9], bladder cancer [10], breast cancer [11], urothelial carcinoma [12,13], and other malignancies. Nevertheless, objective response rates seldom exceed 40% in most tumour types [14,15]. Owing to primary and acquired resistance, a large proportion of patients fail to benefit from, and only a minority respond durably to, anti-PD-1/PD-L1 therapy [16,17]. In other words, the blockade of the PD-1/PD-L1 axis alone is insufficient to normalise the complex tumour immune microenvironment and overcome resistance to PD-1/PD-L1 blockade [18].

Multiple immunosuppressive mechanisms within the tumour microenvironment hinder natural antitumour immunity and limit immunotherapy efficacy [19]. Among these, transforming growth factor-β (TGF-β) plays a central role. Secreted by diverse cell types, all three TGF-βisoforms (TGF-β1, TGF-β2, and TGF-β3) are frequently overexpressed in many cancers and correlate with poor prognosis, metastatic spread, and resistance to therapy [20,21]. TGF-β impairs immune surveillance by suppressing the cytotoxic functions of T cells and natural killer cells, inducing regulatory T cells and myeloid-derived suppressor cells, and inhibiting antigen presentation by dendritic cells [22,23,24]. In parallel, TGF-β promotes tumour progression by driving epithelial–mesenchymal transition (EMT), stimulating angiogenesis [25], and activating cancer-associated fibroblasts (CAFs), which enhance extracellular matrix (ECM) deposition and form physical barriers to immune cell infiltration [26,27,28].

This TGF-β-driven immunosuppressive environment has been associated with resistance to PD-1/PD-L1 blockade, and tumours from resistant patients often exhibit elevated TGFβ1 transcription [26,29]. Co-inhibition of TGF-β and PD-1/PD-L1 signalling has been shown to facilitate T cell penetration into tumours [27,30], suggesting that dual inhibition may overcome resistance [30,31,32].

Several bifunctional antibodies targeting PD-(L)1 and TGF-β such as SHR-1701, YM101 and JS201 are currently undergoing clinical evaluation at various stages. One such agent, the PD-L1/TGF-β fusion antibody M7824, showed limited clinical success and failed to meet key endpoints such as improved progression free survival in multiple late-phase trials [32]. A recent meta-analysis suggests that while safety profiles are comparable, PD-1 antibodies may offer superior efficacy compared to PD-L1 inhibitors [33]. This is likely due to their ability to block both PD-L1 and PD-L2 interactions with PD-1 and provide broader inhibition of immune-evasion pathways. In addition, tumour-derived exosomes enriched in PD-L1 can sequester PD-L1 antibodies before they reach the tumour bed [34]. By directly targeting T cells, PD-1 antibodies may therefore retain greater functional potency within the tumour microenvironment.

To elucidate the mechanistic basis of dual PD-1/TGF-β pathway inhibition, we engineered 015s, a bifunctional fusion antibody that simultaneously blocks murine PD-1 and TGF-β signalling. Antibody 015s consist of a human/mouse cross-reactive anti-PD-1 monoclonal antibody with an mIgG2a-LALA Fc domain, fused to the extracellular domain of human TGF-β receptor II, thereby forming a high-affinity TGF-β trap. Here, we characterise the biochemical properties of 015s, evaluate its antitumour efficacy in murine models, and dissect the mechanisms underlying its therapeutic activity.

## 2. Results

### 2.1. Analysing 015s: A Bifunctional Fusion Antibody That Simultaneously Blocks Murine PD-1 and TGF-β Signalling

**Figure 1:**
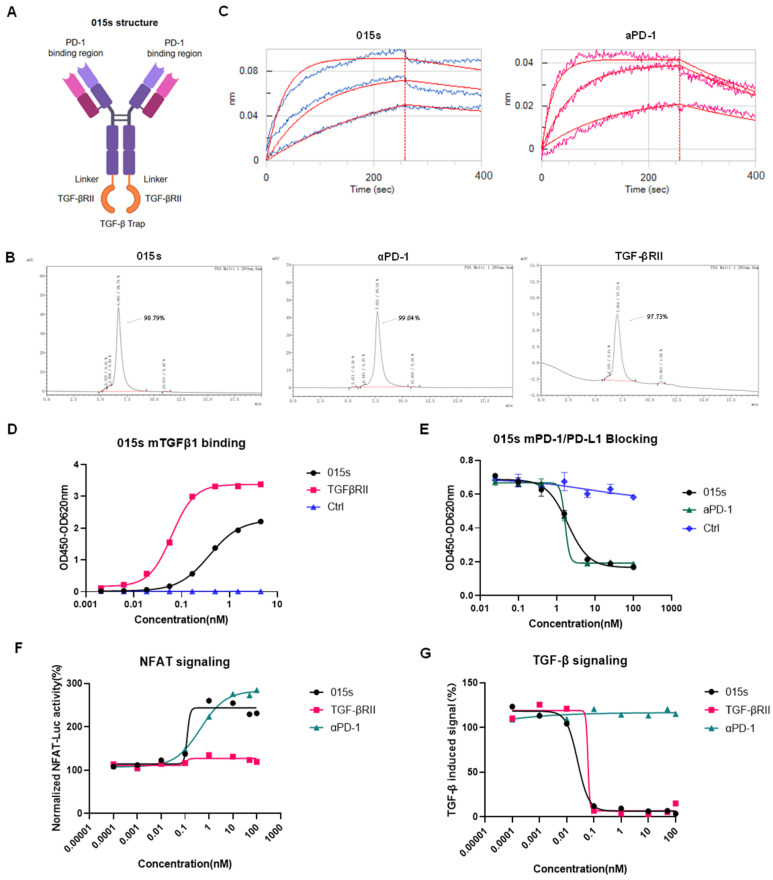
Antibody 015s binds PD-1 and TGF-β with high specificity in vitro, activates T cells, and inhibits TGF-β signalling in vitro. (**A**) Schematic representation of 015s, a bifunctional fusion antibody comprising a human/mouse cross-reactive anti-PD-1 mAb bearing an mIgG2a-LALA Fc domain, fused to the extracellular domain of human TGF-βRII to form a TGF-β trap. (**B**) Size-exclusion HPLC profiles of 015s, anti-PD-1, and TGF-βRII. Protein purity was determined based on the percentage of the main peak at its respective retention time. The x-axis shows retention time (min), and the y-axis shows absorbance (mAU). (**C**) Bio-layer interferometry binding kinetics of 015s and anti-PD-1 to recombinant mouse PD-1. Equilibrium dissociation constants (KD) were calculated using a 1:1 binding model (Octet software). The fluctuating line represents the raw detection signal, whereas the smooth line denotes the fitted curve. (**D**) ELISA showing concentration-dependent binding of 015s and TGF-βRII to plate-immobilised mTGF-β1 (n = 2). (**E**) Competitive ELISA evaluating the ability of 015s and anti PD-1 to inhibit the interaction between mPD-1 and mPD-L1. Serial dilutions of samples were pre-incubated with mPD-1 prior to application to mPD-L1-coated plates (n = 2). (**F**) Jurkat-hPD-1-NFAT-Luc was co-cultured with CHO-K1-OKT3-hPD-L1 cells at a 2:1 ratio for 6 h in the presence of serial dilutions of 015s, anti-PD-1, or TGF-βRII. Luciferase activity (RLU) was measured and normalised to maximum signal (n = 3). (**G**) HEK-293T-SEB-Luc cells were treated with graded concentrations of 015s, anti-PD-1, or TGF-βRII in the presence of recombinant human TGF-β1 (10 ng/mL) for 16 h. Luciferase activity was used to quantify TGF-β signalling inhibition (n = 3).

Antibody 015s is a bifunctional fusion antibody created by fusing a human/mouse cross-reactive anti-PD-1 monoclonal antibody with the extracellular domain of human TGF-β receptor II (TGF-βRII), thereby enabling simultaneous blockade of the PD-1/PD-L1 axis and soluble TGF-β (Figure 1A). Size-exclusion HPLC confirmed that 015s, anti-PD-1, and TGF-βRII were each >95% pure (Figure 1B). Bio-layer interferometry showed that 015s retained high affinity for mouse PD-1 (1.38 × 10^−9^ M), comparable to its parental anti-PD-1 antibody (3.77 × 10^−9^ M), indicating that fusion to TGF-βRII does not compromise PD-1 binding (Figure 1C). Based on ELISA, both 015s and TGF-βRII bound plate-immobilised mTGF-β1 with EC_50_ values of 0.3786 nM and 0.06193 nM, respectively (Figure 1D). Competitive ELISA demonstrated that 015s inhibited the mPD-1/mPD-L1 interaction in a concentration-dependent manner (IC_50_ = 1.91 nM; Figure 1E). In a Jurkat-hPD-1/NFAT-luciferase-reporter assay, 015s, but not TGF-βRII, activated NFAT signalling in a dose dependent manner with an EC_50_ of 0.1136 nM (Figure 1F). Conversely, 015s, but not anti-PD-1, potently suppressed TGF-β signalling in a HEK293-SEB-luc SMAD reporter assay (IC_50_ = 0.02511 nM; Figure 1G). Collectively, these results show that 015s preserves high-affinity binding to both targets and concurrently inhibits the PD-1/PD-L1 and TGF-β pathways.

### 2.2. Antibody 015s Antagonises TGF-β-Induced Epithelial–Mesenchymal Transition and Suppresses Tumour Cell Migration In Vitro

**Figure 2:**
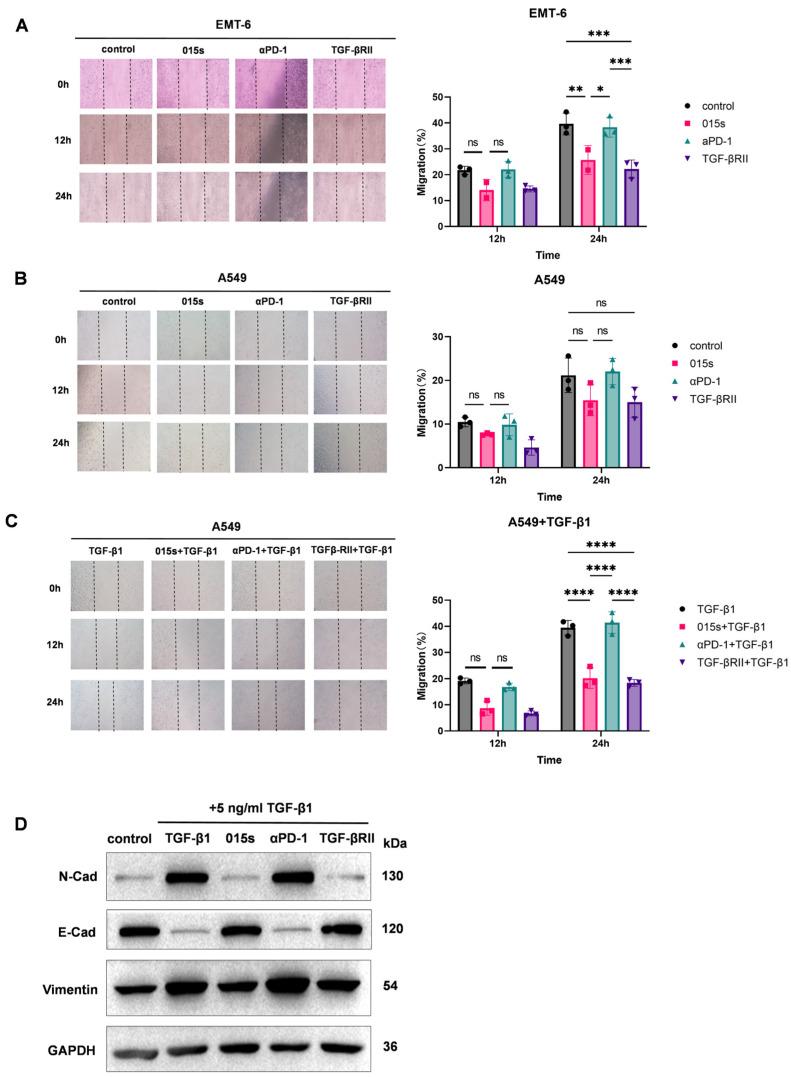
Antibody 015s inhibits tumour cell migration and TGF-β-induced epithelial–mesenchymal transition in vitro. (**A**–**C**). Wound-healing assays were performed on EMT-6 (**A**) and A549 (**B**,**C**) cells treated with 015s, parental anti-PD-1, TGF-βRII, or combinations thereof in the absence or presence of TGF-β1 (5 ng/mL). Representative images (×100) were captured at 0, 12, and 24 h post scratch. Migration was quantified as the percentage of wound closure relative to 0 h (right panels). Data are mean ± SEM (n = 3). NS, not significant (*p* > 0.05); * *p* < 0.05; ** *p* < 0.01; *** *p* < 0.001; **** *p* < 0.0001. (**D**) Western blot analysis of EMT markers in A549 cells treated with TGF-β1 (5 ng/mL) and 015s, anti-PD-1, or TGF-βRII for 48 h. Expression of N-cadherin, E-cadherin, and Vimentin was assessed, and GAPDH served as a loading control.

We performed scratch-wound assays using two independent cell lines, EMT-6 and A549, and found that 015s, but not the parental anti-PD-1 antibody, significantly reduced wound closure at 24 h, indicating impaired migratory capacity (Figure 2A,B). In EMT-6 cells, treatment with 015s led to a significant reduction in migration at 24 h compared to control (*p*  <  0.01), while anti-PD-1 had no significant effect. Similarly, in A549 cells, 015s significantly impaired migration only when cells were stimulated with exogenous TGF-β1 (*p*  <  0.0001 compared to TGF-β1 alone; Figure 2C), confirming that the TGF-β-trapping ectodomain fused to its heavy chain remains functionally active and efficiently neutralises TGF-β. Western blot analysis confirmed that TGF-β1 treatment induced EMT, as evidenced by increased Vimentin and N-cadherin expression and reduced E-cadherin levels. Treatment with 015s reversed these molecular changes, markedly attenuating TGF-βinduced EMT (Figure 2D). Together, these findings demonstrate that 015s counteracts TGF-β-mediated EMT and consequently restricts tumour cell migration in vitro.

### 2.3. Antibody 015s Demonstrates Superior Antitumour Efficacy in An Orthotopic EMT-6 Murine Breast Cancer Model

**Figure 3:**
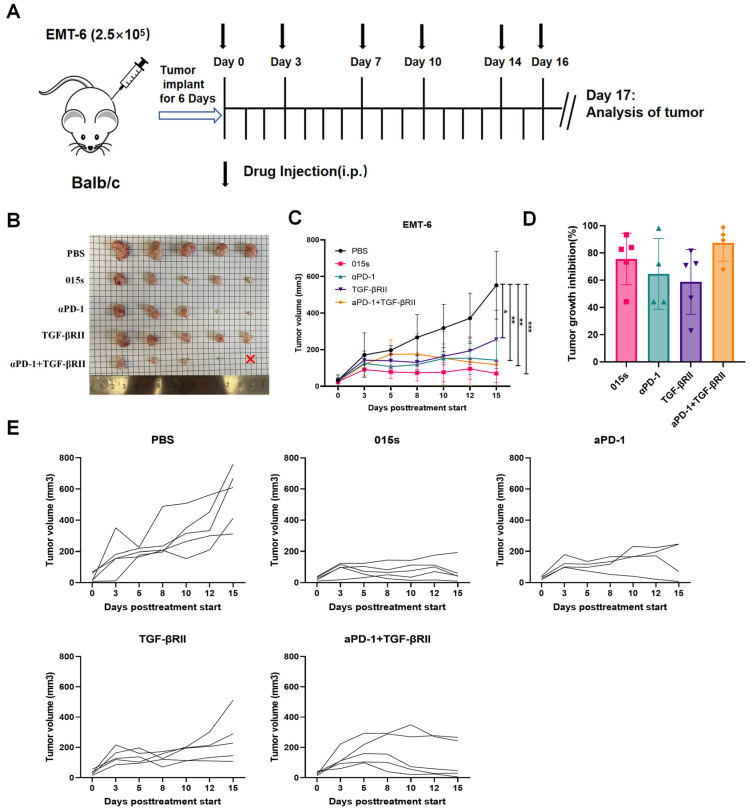
Antibody 015s markedly inhibits tumour growth in an orthotopic EMT-6 breast tumour model. BALB/c mice were orthotopically implanted with 2.5 × 10^5^ EMT-6 cells into the right mammary fat pad. Treatment commenced once tumours reached 50–100 mm^3^ in volume, and tumour volumes were measured every 1–2 days. Mice were euthanised on day 17, one day after the final dose was administered. (**A**) Experimental timeline and intraperitoneal dosing schedule; 015s was administered at 10 mg/kg every 2–3 days for a total of six doses. Equimolar comparator treatments included anti-PD-1 (αPD-1, 8.1 mg/kg), TGF-βRII (9.6 mg/kg), or a combination of αPD-1 (8.1 mg/kg) + TGF-βRII (4.8 mg/kg) administered with the same schedule. (**B**) Photographs of all excised tumours at endpoint. The red × denotes an animal in the combination group that died unexpectedly prior to tumour collection. (**C**) Mean tumour volume over time. (**D**) Tumour growth inhibition at endpoint. (**E**) Individual tumour growth curves for each mouse. Data represent mean ± SEM (n = 5). * *p* < 0.05; ** *p* < 0.01; *** *p* < 0.001.

We evaluated the antitumour activity of 015s in comparison to anti-PD-1 (αPD-1), soluble TGF-βRII, and the αPD-1 + TGF-βRII combination in BALB/c mice bearing orthotopic EMT-6 mammary tumours (Figure 3A). While monotherapy with either αPD-1 or TGF-βRII led to only modest tumour growth inhibition, treatment with 015s resulted in significantly greater tumour suppression compared to equimolar doses of either αPD-1 or TGF-βRII alone (*p* < 0.01, Figure 3B–E). In the combination cohort, one animal, which had the largest tumour, died unexpectedly the day prior to tumour excision. Because the tumour could not be harvested, this introduced a minor discrepancy in the growth curves on day 15 (relative to the treatment start day) and in the inhibition rates for that group. After accounting for this limitation, the overall antitumour activity of 015s remained comparable to that of the αPD-1 + TGF-βRII combination, with no statistically significant difference observed between the two treatment groups (*p* > 0.05).

These results indicate that 015s offers a therapeutic efficacy at least equivalent to combined PD-1 and TGF-β pathway blockade, while delivering both activities in a single agent.

### 2.4. Antibody 015s Reduces Systemic and Intratumoural TGF-β1 While Elevating Cytotoxic Cytokines

**Figure 4:**
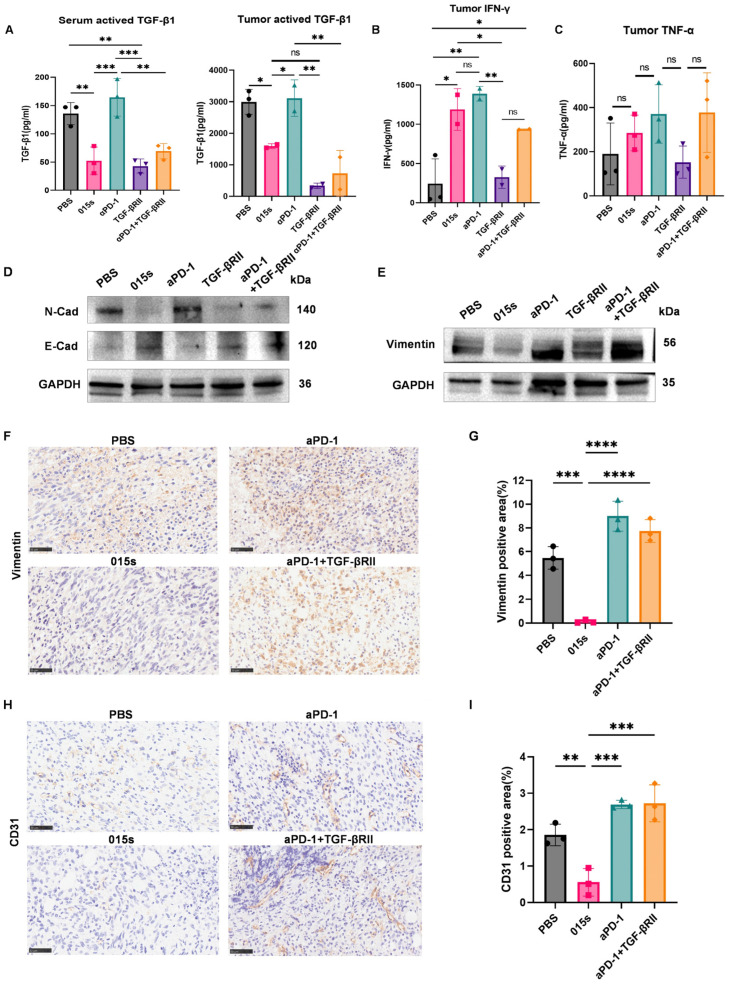
Antibody 015s reduces TGF-β1 levels, enhances intratumoural cytokine production, reverses tumour cell EMT, and limit angiogenesis in the EMT-6 tumour model. Circles represent the PBS group, squares represent the 015 s group, upward-pointing triangles represent the aPD-1 group, downward-pointing triangles represent the TGF-βRII group and diamonds represent the aPD-1+TGF-βRII group. (**A**–**C**) Cytokine levels in serum and tumour homogenates were quantified by ELISA. (**A**) Active TGF-β1 quantified in undiluted serum and 5-fold-diluted tumour supernatants using pre-coated capture antibody plates. (**B**) IFN-γ measured in 10-fold-diluted tumour supernatants. (**C**) TNF-α measured in 5-fold-diluted tumour supernatants. (**D**,**E**) Western blot analysis of EMT markers E-cadherin, Vimentin, and N-cadherin with GAPDH as a loading control in EMT-6 tumours. (**F**–**I**) Immunohistochemistry (IHC) of EMT and angiogenesis markers in EMT-6 tumours (400×; scale bar: 50 µm). (**F**,**G**) Representative images of Vimentin IHC and quantification of Vimentin-positive tumour area. (**H**,**I**) Representative images of CD31 IHC and quantification of CD31-positive area as a measure of tumour vascularisation. Data are presented as mean ± SD (n = 3); ns, not significant (*p* > 0.05); * *p* < 0.05; ** *p* < 0.01, *** *p* < 0.001, and **** *p* < 0.0001. Circles represent the PBS group, squares represent the 015 s group, triangles represent the aPD-1 group and diamonds represent the aPD-1+TGF-βRII group.

Cytokine analysis by ELISA demonstrated that 015s significantly reduced levels of active TGF-β1 in both serum and tumour tissue compared to PBS, αPD-1, and the αPD-1 + TGF-βRII combination (*p*  <  0.01–0.001) to a degree comparable to the parental TGF-βRII trap (Figure 4A). Conversely, 015s, but not TGF-βRII, significantly increased intratumoural IFN-γ (*p*  <  0.05 vs. PBS and *p*  <  0.01 vs. TGF-βRII), resembling the effect of αPD-1 (Figure 4B). TNF-α levels concentrations exhibited high intra-group variability, with the highest mean level in the 015s group, although differences were not statistically significant (Figure 4C). Collectively, these data indicate that 015s penetrates tumour tissue, neutralises TGF-β1-mediated immunosuppression, and reinstates a pro-inflammatory, cytotoxic milieu, thereby contributing to its antitumour efficacy.

### 2.5. Antibody 015s Reverses Epithelial–Mesenchymal Transition and Suppresses Angiogenesis in the EMT-6 Murine Breast Tumour Model

To confirm that 015s neutralises TGF-β signalling in vivo, we analysed EMT markers. Western blot analysis revealed that 015s reduced expression of Vimentin and N-cadherin and restored protein levels of E-cadherin (Figure 4D,E). These changes were supported by IHC analysis of Vimentin, where 015s-treated tumours showed a significant reduction in Vimentin-positive area compared to PBS (*p*  <  0.001), αPD-1 (*p*  <  0.001), and αPD-1 + TGF-βRII (*p*  <  0.0001; Figure 4F,G). Similarly, CD31 staining showed lower microvascular density in the 015s group compared to all other groups (*p*  <  0.01–0.001), indicating a marked anti-angiogenic effect (Figure 4H,I). These findings demonstrate that 015s curtails tumour progression by simultaneously inhibiting EMT and tumour angiogenesis.

Given the central role of TGF-β in fibroblast activation and extracellular matrix deposition, we next assessed whether 015s also attenuates cancer-associated fibroblast activity and tumour fibrosis.

### 2.6. Antibody 015s Attenuates Cancer-Associated Fibroblast Activity and Mitigates Collagen Deposition Within Tumours by Downregulating α-SMA

**Figure 5:**
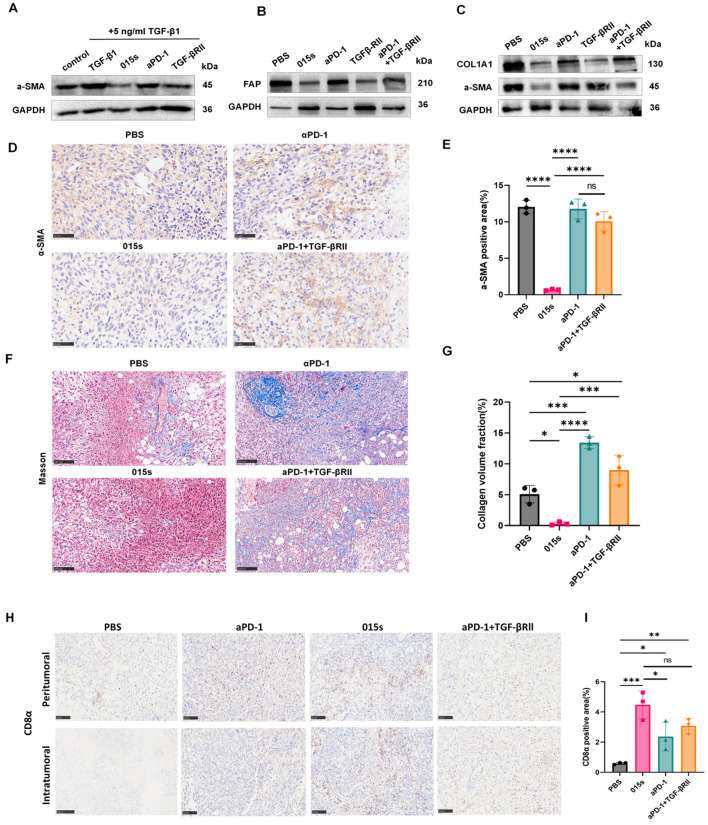
Antibody 015s reduces α-SMA and collagen deposition, curtails CAF activity, and mitigates tumour fibrosis. (**A**) In vitro assessment of myofibroblast differentiation. MRC-5 fibroblasts were stimulated with TGF-β1 (5 ng/mL) and treated for 48 h with 015s, anti-PD-1 or TGF-βRII. Total protein lysates were analysed by Western blotting for α-SMA to evaluate the TGF-β-induced fibroblast-to-myofibroblast transition. (**B**,**C**) Western blot analysis of EMT-6 tumour lysates for CAF markers α-SMA and FAP (**B**), and ECM marker COL1A1 (**C**). (**D**,**E**) Representative IHC images for α-SMA (**D**) with quantification of α-SMA-positive area (**E**). (**F**,**G**) Masson’s trichrome staining for collagen fibres (**F**) and quantification of collagen volume fraction (**G**). (**H**,**I**) Representative images and quantitative analysis of anti-CD8α IHC in the mouse breast cancer model of EMT-6 (400×; scale bar: 50 µm). The results for each group are shown as mean ± SD (n = 3). ns, not significant (*p* > 0.05); * *p* < 0.05, ** *p* < 0.01, *** *p* < 0.001, and **** *p* < 0.0001. Circles represent the PBS group, squares represent the 015 s group, triangles represent the aPD-1 group and diamonds represent the aPD-1+TGF-βRII group.

In vitro Western blot analysis confirmed that 015s, but not αPD-1, antagonised TGF-β-induced upregulation of α-SMA, thereby inhibiting the MRC-5 to myofibroblast transition (Figure 5A). To determine whether 015s similarly alleviates tumour fibrosis in vivo, we evaluated α-SMA, FAP, and COL1A1 expression in EMT-6 tumours by Western blotting. Compared with vehicle and αPD-1 monotherapy, 015s markedly reduced all three fibrosis markers (Figure 5B,C). Consistent with the Western blot findings, IHC revealed a significant reduction in α-SMA-positive area in the 015s treatment group compared with PBS, αPD-1, and αPD-1 + TGF-βRII (*p*  <  0.0001; Figure 5D,E), indicating that the TGF-βRII arm of 015s is the principal mediator of this effect. Masson’s trichrome staining further demonstrated that 015s substantially diminished collagen deposition, as shown by a significantly lower collagen volume fraction compared to all other groups (*p* < 0.05–0.0001; Figure 5F,G). In contrast, αPD-1 monotherapy increased collagen accumulation relative to PBS (*p*  <  0.001), suggesting that blockade of PD-1 alone may exacerbate tumour fibrosis.

Masson’s trichrome staining showed that 015s markedly reduced aniline-blue-positive collagen fibres in EMT-6 tumours, indicating diminished stromal deposition secondary to lower CAF activity (Figure 5F,G). In contrast, anti-PD-1 monotherapy produced a denser, coarser collagen network than the vehicle control, suggesting that PD-1 blockade alone may actually intensify tumour fibrosis. Because excessive fibrosis can impede T cell penetration, we next examined CD8α immunohistochemistry. In the αPD-1 group, CD8^+^ T cells were confined largely to the tumour periphery, whereas 015s drove robust infiltration into the tumour core, coinciding with a stronger antitumour effect (Figure 5H,I). Together, these findings indicate that 015s alleviates tumour fibrosis and, in turn, facilitates deep CD8^+^ T cell access to the tumour parenchyma.

### 2.7. Antibody 015s Combined with an ADC Induces Robust Tumour Regression in a Mouse Model

Immune checkpoint inhibitors, particularly PD-1/PD-L1 antibodies, have shown potent therapeutic synergy with antibody–drug conjugates (ADCs) in recent clinical studies. ADCs that carry tubulin or topoisomerase inhibitors can elicit both innate and adaptive immune responses, markedly increasing T cell infiltration into tumours and thereby augmenting the activity of checkpoint blockade. Conversely, checkpoint inhibitors have been reported to enhance the tumour-eradicating capacity of ADCs [35].

cG7-MMAE is an in-house ADC candidate in which an anti-human CD24 monoclonal antibody (cG7) is conjugated to the microtubule inhibitor monomethyl auristatin E (MMAE). The CD24-specific antibody component activates macrophages, whereas the MMAE payload induces G_2_/M arrest and apoptosis, effectively killing cancer cells [35].

**Figure 6:**
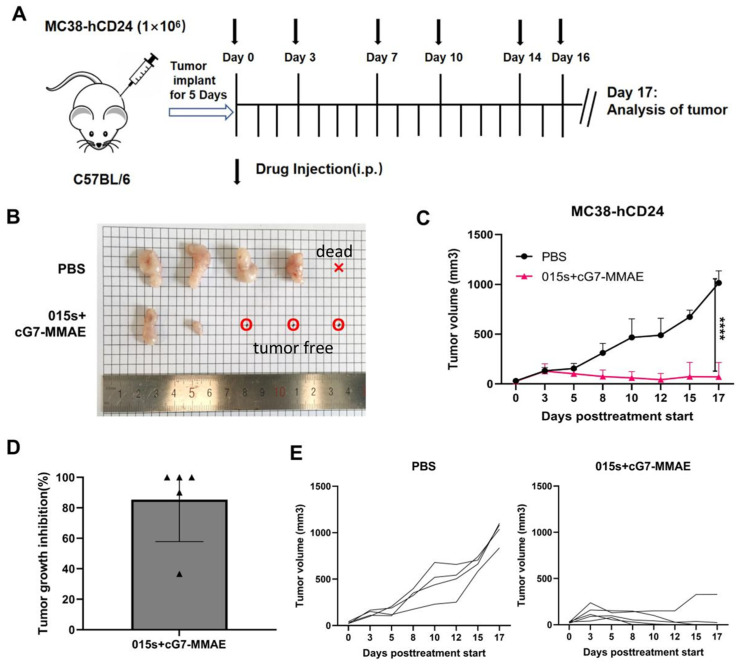
The combination of 015s and ADC drugs demonstrated superior antitumour activity. Tumour volume of mice was measured every one to two days. The day after the end of dosing, the mice were euthanized. (**A**) Establishment of MC38-hCD24 tumour model and treatment plan. The injection dose of 015s is 10 mg/kg, and the injection dose of the ADC drug cG7-MMAE is 0.5 mg/kg. The dosing sequence was intraperitoneal injection of cG7-MMAE, followed by 015s after an interval of half an hour and administered every 2 or 3 days, a total of 6 times. (**B**–**E**) On day 0, 1 × 10^6^ MC38-hCD24 cells were inoculated subcutaneously in the right axillary arm of C57BL/6 mice. Treatment started on day 6. The representative images of tumours, tumour growth curves, tumour inhibition rates, and tumour growth curve data for each mouse in each dosing group of mice treated with 015s + cG7-MMAE and controls are shown. The results for each group are shown as mean ± SD (n = 3). **** *p* < 0.0001.

We evaluated the antitumour efficacy of 015s in combination with cG7-MMAE in the MC38-hCD24 murine colorectal cancer model (Figure 6A). Compared with the vehicle control group, the combination therapy significantly inhibited tumour growth (*p*  <  0.0001) and induced pronounced tumour regression, with three out of five treated mice exhibiting complete tumour eradication (Figure 6B–E). These findings provide compelling pre-clinical evidence that dual PD-1/TGF-β blockade with 015s can cooperate with ADC therapy to achieve superior antitumour outcomes.

## 3. Discussion

The advent of immune-checkpoint inhibitors, exemplified by antibodies against PD-1/PD-L1, has revolutionised oncology. Yet, numerous studies have shown that these agents are significantly less effective against immunologically “cold” tumours [36]. Thus, converting cold tumours into “hot” tumours, alleviating the immunosuppressive tumour microenvironment, and thereby heightening sensitivity to PD-1/PD-L1 blockade remain urgent clinical challenges [19,36]. TGF-β is a pleiotropic cytokine whose tumour-promoting or tumour-suppressive activity depends on cancer type and disease stage [25]. In advanced disease, it typically shifts from a tumour-suppressive to a tumour-promoting role [23]. A TGF-β-rich TME fosters metastasis, promotes epithelial–mesenchymal transition, and impairs immune-cell function, thereby facilitating immune escape [27,37]. Activation of the fibroblast TGF-β signalling pathway further drives stromal remodelling and collagen deposition, which impedes immune cell infiltration, rendering the tumour immunologically cold [20,38]. Targeting TGF-β signalling is therefore a promising strategy to restore antitumour immunity and overcome resistance to immune checkpoint blockade. In view of the cooperative role of the PD-1/PD-L1 axis and TGF-β pathway in immune evasion, we developed 015s which is a bifunctional fusion antibody designed to simultaneously both signalling axes.

Bio-Layer Interferometry and ELISA analyses confirmed that 015s binds murine PD-1 and TGF-β with high affinities comparable to its parental components. Functionally, 015s acts as a TGF-β trap: like soluble TGF-βRII, it neutralises TGF-β bioactivity and blocks downstream signalling. In vitro, 015s reversed TGF-β-induced EMT, thereby suppressing tumour cell migration, while its PD-1 targeting arm restored T cell signalling.

In the EMT-6 orthotopic breast tumour model, 015s demonstrated superior efficacy compared to either TGF-βRII or anti-PD-1 monotherapy. ELISA revealed that 015s markedly reduced active TGF-β1 in serum and tumour tissue while significantly increasing intratumoural cytokines, such as TNFα and IFN-γ. Western blotting and IHC analyses confirmed that 015s decreased Vimentin, N-cadherin and CD31 while restoring E-cadherin, which is consistent with a reversal of EMT and a reduction in angiogenesis.

Previous work indicates that EMT-6 tumours, characterised by high TGF-β levels, respond poorly to PD-1/PD-L1 inhibitors due to T cell exclusion mediated by CAF-driven stromal barriers [26]. TGF-β activation in CAF enhances collagen production and extracellular matrix (ECM) remodelling, further impending immune infiltration [26]. Consistent with this, we found that 015s, and to a lesser extent TGF-βRII, reduced expression of α-SMA, FAP, and COL1A1. These changes were accompanied by a reduced collagen volume fraction, indicating alleviation of tumour fibrosis and reduced CAF activity [39,40,41]. Importantly 015s also increased CD8^+^ T cell infiltration into the tumour core, suggesting conversion from an immune-excluded phenotype to an inflamed phenotype. Given the pro-metastatic role of CAFs, these effects imply that 015s may also limit metastatic potential by restraining CAF-driven stromal remodelling. Finally, combining 015s with the CD24-targeted ADC cG7-MMAE yielded superior antitumour efficacy in the MC38-hCD24 colorectal model, providing initial proof that dual PD-1/TGF-β blockade can cooperate with ADC therapy in cancer treatment.

Nonetheless, this study has limitations. Blockade of TGF-β signalling is expected to affect other immunosuppressive compartments (e.g., Tregs, MDSCs, and myeloid populations), and we will profile these changes using single-cell RNA-seq and multiparameter flow cytometry in future work. Likewise, although combining 015s with the CD24-targeted ADC cG7-MMAE improved tumour control, a formal demonstration of synergy will require inclusion of the corresponding monotherapy arms; these controls will be incorporated into subsequent studies.

## 4. Materials and Methods

### 4.1. Cell Lines and Antibodies

Murine mammary carcinoma EMT-6, human lung carcinoma A549, and human embryonic lung fibroblast MRC-5 cell lines were obtained from the American Type Culture Collection (ATCC). HEK293-SEB-Luc, Jurkat-PD-1/NFAT, and CHO-K1/OKT3/PD-L1 reporter cell lines were provided by Nanjing Leads Biolabs Co., Ltd. (Nanjing, China).

EMT-6 and A549 cells were maintained in RPMI-1640 (Gibco) supplemented with 10% foetal bovine serum (FBS, ExCell). Jurkat-PD-1/NFAT cells were cultured in RPMI-1640 containing 400 µg/mL hygromycin B, 500 µg/mL G418, and 10% FBS. CHO-K1/OKT3/PD-L1 cells were grown in DMEM/F-12 medium supplemented with 4 µg/mL puromycin, 600 µg/mL G418, and 10% FBS. MRC-5 was cultured in MRC-5 medium-specific cells (Procell) containing 10% FBS., whereas HEK293-SEB-Luc cells were cultured in DMEM (Gibco) plus 10% FBS. All cultures were kept at 37 °C in a humidified incubator with 5% CO_2_.

The bifunctional fusion antibody 015s (anti-PD-1/TGF-β), anti-PD-1 monoclonal antibody, soluble TGF-β receptor II (TGF-βRII), and isotype control antibody were expressed and purified by Nanjing Leads Biolabs Co., Ltd. (Nanjing, China).

### 4.2. Size-Exclusion High-Performance Liquid Chromatography (SEC-HPLC)

Protein purity of 015s, anti-PD-1, and TGF-βRII was assessed on an LC-20 HPLC system (Shimadzu, Kyoto, Japan) equipped with a TSKgel G3000SWXL column (Tosoh, Tokyo, Japan). After column equilibration with 25 mM phosphate-buffered saline (PBS) at a flow rate of 1 mL min^−1^, 20 min runs were performed at 25 °C with UV detection at 280 nm. Samples were diluted in PBS and injected via an autosampler, and purity was determined from peak shape and retention time.

### 4.3. Bio-Layer Interferometry

Binding kinetics of 015s and anti-PD-1 to mPD-1 were measured using an Octet RED96 (ForteBio, CA, USA). Anti-human Fc capture (AHC) sensors were pre-hydrated and equilibrated in running buffer (0.02% Tween-20 in PBS). mPD-1 (100 nM, Sino Biological, Beijing, China) was loaded for 200 s, followed by baseline acquisition (300 s). Antibody concentrations ranged from 6.3 nM to 50 nM in a threefold serial dilution. Association (200 s) and dissociation (600 s) phases were recorded, and sensors were regenerated in 0.01 M glycine-HCl (pH 2.0) for 30 s. Data were fitted with a 1:1 binding model using Data Analysis HT (Sartorius, Göttingen, Germany).

### 4.4. Enzyme-Linked Immunosorbent Assay

The binding activities of 015s and soluble TGF-βRII to mTGF-β1 were determined by a standard indirect ELISA. Recombinant mTGF-β1 (Sino Biological) was diluted to 0.2 µg/mL and dispensed at 50 µL per well into 96-well plates (BIOFIL, Guangzhou, China), followed by overnight coating at 4 °C. Plates were washed three times with wash buffer (0.05% Tween-20 in PBS) and blocked with PBS containing 1% bovine serum albumin (BSA) for 1 h at 37 °C. Serial dilutions of 015s, TGF-βRII, or isotype control (50 µL per well) were then added and incubated for 1 h at 37 °C. After four washes, bound antibody was detected with goat anti-human IgG Fc (HRP; 1:20,000, Jackson ImmunoResearch, West Grove, PA, USA) for 1 h at 37 °C. Following additional washes, TMB (Thermo Scientific, MA, USA) was added, and the reaction was terminated with 1 M H_2_SO_4_. Absorbance was measured at 450 nm with background correction at 620 nm using a Tecan microplate reader.

The ability of 015s and an anti-PD-1 monoclonal antibody to inhibit the interaction between mPD-1 and mPD-L1 was assessed by competitive ELISA. mPD-L1 (Sino Biological) was diluted to 2 µg/mL, plated at 50 µL per well, and coated overnight at 4 °C. After washing and blocking as described above, graded concentrations of 015s, anti-PD-1, or control (50 µL per well) were mixed 1:1 (v/v) with mPD-1 (4 µg/mL final) and added to the plate for 1 h at 37 °C. Plates were washed four times and incubated for 30 min at room temperature with goat anti-human IgG Fc-HRP (1:10,000, Jackson ImmunoResearch). Colour development and data acquisition were performed as described for the binding ELISA.

Cytokine levels in serum and tumour tissues from EMT-6 tumour-bearing mice were quantified with pre-coated ELISA kits. Retro-orbital blood collection was performed under isoflurane anaesthesia in accordance with institutional animal care guidelines and allowed to clot for 2 h at room temperature and centrifuged (3000 rpm, 10 min, 4 °C) to obtain serum. Fresh or liquid-nitrogen-frozen tumour samples were mixed with PBS (1:5, w/v) and homogenised in a 3D cryogenic grinder (Servicebio, Wuhan, China), and supernatants were collected after centrifugation. TGF-β1, IFN-γ, and TNF-α concentrations were quantified using mouse-specific ELISA kits (DAKEWE, Shenzhen, China) according to the manufacturer’s instructions. Absorbance was measured at 450 nm with a 620 nm reference using a BioTek microplate reader.

### 4.5. TGF-β/SMAD Luciferase Reporter Assay in HEK293-SEB-Luc Cells

HEK293-SEB-Luc cells (1 × 10^4^ cells/well) were seeded in 96-well plates and treated the following day with graded concentrations of 015s, anti-PD-1, or TGF-βRII in the presence of 10 ng/mL TGF-β1 (Abclonal). After 16 h, luciferase activity was measured using a Luciferase Assay Kit (Yeasen).

### 4.6. NFAT Luciferase Reporter Assay

Jurkat-PD-1/NFAT (1 × 10^5^ cells/well) and CHO-K1/OKT3/PD-L1 (0.5 × 10^5^ cells/well) cells were co-cultured in 96-well plates with serial dilutions of 015s, anti-PD-1, or TGF-βRII. After 6 h, cells were lysed, and luciferase activity was quantified (Yeasen).

### 4.7. Wound-Healing Assay

EMT-6 or A549 cells were grown to 80–90% confluence in 24-well plates, serum-starved (2% FBS), and scratched with a 10 µL pipette tip. Cells were treated with 100 nM 015s, anti-PD-1, or TGF-βRII in the presence or absence of 5 ng/mL TGF-β1. Wound closure was photographed at 0, 12, and 24 h. Migration rate (%) was calculated as [(initial area−remaining area)/initial area] × 100%. Data were analysed using ImageJ (version 1.8.), and data were plotted in GraphPad Prism (version 8).

### 4.8. Western Blotting

Cells or tumour tissues were lysed in RIPA buffer (Yeasen) and clarified by centrifugation (12,000 rpm, 20 min, 4 °C). Protein concentrations were determined using a BCA protein assay kit (Beyotime, Shanghai, China). Equal amounts of protein were separated by SDS-PAGE and transferred to PVDF membranes (Millipore, Darmstadt, Germany). Membranes were blocked with 5% skim milk and incubated overnight at 4 °C with primary antibodies against N-cadherin (HUABIO, Hangzhou, China, M1304-1), E-cadherin (HUABIO, Hangzhou, China, EM0502), Vimentin (Proteintech, IL, USA, 10366-1-AP), α-SMA (Proteintech, IL, USA, 14395-1-AP), collagen I, FAP, or GAPDH (dilutions 1:2000–1:10,000). HRP-conjugated secondary antibodies (Yeasen, Shanghai, China) were applied for 1 h at room temperature, and signals were visualised with ECL substrate (Yeasen) on a chemiluminescence imaging system (Tanon, Shanghai, China).

### 4.9. In Vivo Tumour Models

Female BALB/c mice (Yangzhou University) were housed under specific-pathogen-free conditions with ad libitum access to food and water. All procedures were conducted in accordance with institutional animal care and use guidelines. Breast tumours were established by injecting EMT-6 cells (2.5 × 10^5^) into the right mammary fat pad. When tumour volume (TV) reached 50–100 mm^3^ (day 7), mice were randomised (n = 5 per group) to receive PBS, 015s (10 mg/kg), anti-PD-1 (8.1 mg/kg), TGF-βRII (9.6 mg/kg), or anti-PD-1 + TGF-βRII (8.1 + 4.8 mg/kg) via intraperitoneal injection every 2–3 days (six total doses). Tumour size was measured every 1–2 days, and TV was calculated as follows: TV = length × width^2^ × 0.5. Mice were euthanised when TV exceeded 1500 mm^3^ or at the end of the study.

Subcutaneous MC38-hCD24 model: 1 × 10^6^ MC38-hCD24 cells were inoculated subcutaneously in the right axillary arm of C57BL/6 mice. When the average tumour volume reached 50–100 mm^3^, the tumour-bearing mice were divided randomly into groups (5/group): 015s (10 mg/kg) + cG7-MMAE (0.5 mg/kg), PBS.

### 4.10. Immunohistochemistry

Tumours were fixed in 4% paraformaldehyde for 24 h, embedded in paraffin, and sectioned. After de-paraffinisation, sections underwent antigen retrieval in Tris-EDTA (pH 9.0) at 95–100 °C, for 20 min, followed by blocking with 5% goat serum. Slides were incubated overnight at 4 °C with primary antibodies against Vimentin (proteintech), α-SMA (proteintech), CD31 (proteintech), or CD8α (Abclonal). HRP-conjugated secondary antibodies were applied, and staining was visualised using diaminobenzidine (DAB). Whole-slide images were acquired using a NanoZoomer S60 scanner (Hamamatsu), and the percentage of tissue area showing positive staining was quantified with ImageJ. Collagen deposition in tumour tissues was evaluated by Masson staining, performed by the Pathology Core of the Target Discovery Centre, China Pharmaceutical University.

### 4.11. Statistical Analysis

All data are presented as mean ± standard deviation (SD) or mean ± standard error of the mean (SEM), as indicated. Statistical analyses were performed using GraphPad Prism (GraphPad Software, San Diego, CA, USA). Group comparisons were made using one-way ANOVA. For pairwise comparisons, Student’s t-test (unpaired, two-tailed) was used. *p*-values < 0.05 were considered statistically significant.

## 5. Conclusions

In summary, 015s normalises the immunosuppressive tumour microenvironment by simultaneously blocking two key inhibitory pathways, PD-1/PD-L1 and TGF-β, thereby restoring antitumour immunity and demonstrating potent efficacy in murine models. These proof-of-concept data support further development of a humanised PD-1/TGF-β bifunctional fusion antibody, which could offer a novel therapeutic option for patients with tumours refractory to current immune checkpoint inhibitors. Moreover, such bifunctional molecules could be combined with other therapeutic modalities, antibody–drug conjugates (ADCs), small-molecule agents, or additional biologics, to target multiple pathways concurrently, remodel the TME through complementary mechanisms, and deliver more robust antitumour responses across a broader patient population.

## Data Availability

The data presented in this study are available on request from the corresponding author due to request.

## Abbreviations

The following abbreviations are used in this manuscript:

PD-1	Programmed cell death protein 1
PD-L1	Programmed death-ligand 1
TGF-β	Transforming growth factor-beta
TGF-βRII	Transforming growth factor-beta receptor II
TME	Tumour microenvironment
CTLA-4	Cytotoxic T-lymphocyte-associated protein 4
EMT	Epithelial–mesenchymal transition
CAF	Cancer-associated fibroblast
ECM	Extracellular matrix
mIgG2a	Mouse immunoglobulin G subclass 2a
SEC-HPLC	Size-exclusion chromatography high-performance liquid chromatography
ELISA	Enzyme-linked immunosorbent assay
MMAE	Monomethyl auristatin E
ADC	Antibody–drug conjugate
